# The hope of children and families in the context of cancer: a scoping review

**DOI:** 10.3389/fped.2026.1958432

**Published:** 2026-10-01

**Authors:** Camila Garófalo, Aline Oliveira Silveira, Dhiovanna Tavares, Hellen Seganfredo, Monika Wernet, Zaida Charepe

**Affiliations:** 1 Graduate Program in Nursing, Faculty of Health Sciences, University of Brasilia, Brasilia, Brazil; 2 Graduate Program in Nursing, Federal University of São Carlos, São Carlos, Brazil; 3 Faculty of Health Sciences and Nursing, Center for Interdisciplinary Research in Health, Universidade Católica Portuguesa, Lisbon, Portugal

**Keywords:** cancer, child, coping skills, family, hope, nursing

## Abstract

**Context:**

Childhood cancer affects children, adolescents, and their families. This sudden and uncertain context threatens hope, a vital, multidimensional, and evolving force.

**Objective:**

To map scientific evidence for the hope of children, adolescents, and families, the meanings, perceptions, and manifestations of hope associated with coping during childhood cancer treatment.

**Method:**

This scoping review was based on the Dufault and Martocchio model and followed Joanna Briggs Institute methodological guidelines. The search was conducted in six databases (PUBMED, CINAHL, LILACS, SCOPUS, PSYCINFO, and SCIELO) in May 2025 and updated in February 2026, returning 4,380 studies.

**Results:**

A total of 31 studies were eligible, most conducted in the United States with a qualitative approach and interviews. The predominance of the maternal perspective was identified. Evidence revealed meanings of hope as “lifeblood” and “ray of light,” undergoing transformations as the expectation of healing was redefined and culminated in hoping for relief from suffering and dignity at the end of life (Cognitive Dimension). Trust and fear coexist (Affective Dimension), but living “day-to-day,” maintaining family rituals and dynamics, and practicing faith help alleviate helplessness (Affiliative and Behavioural Dimension). Meaningful relationships, coping actions, and the ability to redefine healing justify the predominance of the Affiliative, Behavioral, and Cognitive Dimensions.

**Conclusion:**

Hope is a dynamic and metamorphic force that sustains the family in the face of sudden diagnosis and prognostic uncertainties. Relationships with healthcare professionals can amplify or threaten the family's sense of hope. Expanded family representation and as a systemic unit were gaps.

**Systematic Review Registration:**

https://osf.io/atk6v, identifier 10.17605/OSF.IO/ATK6V.

## Introduction

Childhood and adolescent cancer present a constant increase in incidence rates over time, representing a significant public health concern. Abnormal and disordered growth of cells in the body results in forming hematological and solid tumors. The most significant increases are related to leukemias, myeloproliferative and myelodysplastic diseases, lymphomas and reticuloendothelial diseases, CNS neoplasms and other intracranial and intraspinal neoplasms, soft tissue sarcomas and other extraosseous sarcomas, liver tumors, germ cell tumors, trophoblastic tumors, and gonadal neoplasms. The latency period of the disease is generally short, but with rapid progression, which requires accurate and early diagnosis and treatment (1).

Epidemiological data corroborate the complexity of this situation. An analysis of epidemiological behavior over 45 years has shown an increase in the incidence of childhood cancer, as well as a reduction in mortality and a significant improvement in survival rates in recent years. These advances reveal an increase in survival by 5 and 10 years for some pediatric neoplasms, resulting from improved therapeutic techniques in response to these diagnoses (2).

Nevertheless, inequalities related to sociodemographic factors such as sex, ethnicity, and better access to health services remain with regard to mortality, survival, and the persistence of unfavorable prognoses. This scenario reinforces the fact that despite diagnostic and therapeutic advances, childhood cancer remains a life-threatening condition (2, 3).

The sudden diagnosis and the complex and uncertain course affect the child and family profoundly and comprehensively, producing an emotional imbalance from the diagnostic communication to the uncertainties inherent in the treatment. This scenario requires multiple readjustments throughout the family dynamic, which consequently adds the fear of losing control over their lives and their own future (3, 4).

The treatment of childhood cancer involves a significant accumulation of diverse physical and emotional burdens, characterized by challenging and complex experiences which require continuous and multifaceted coping strategies (3). For example, frequent decision-making regarding therapeutic conduct, reorganizing the routine and family dynamics, managing symptoms and adverse effects of treatment, in addition to the constant emotional demands on the child and family unit. Opposing feelings such as discouragement and hope often connect and coexist, as do fear, anxiety, stress, and pain (both physical and emotional), thus composing the experience of illness due to cancer in the child and adolescent context (3, 4).

In this regard, hope is intrinsic to human beings and emerges as a multidimensional phenomenon and construct represented by diverse meanings and characterized by a confident, albeit uncertain, expectation. It is a constantly evolving phenomenon, permeated by thoughts, affections, and actions which transform throughout the coping process (5, 6). Hope, as a coping element, plays a significant role in the pediatric oncology setting, supporting children, adolescents, and families in the face of adversity, promoting meaning, involvement, and resilience throughout treatment (5).

According to the Dufault and Martocchio model (5), hope is composed of two spheres, generalized and particularized, and six dimensions: affective, cognitive, behavioral, affiliated, temporal, and contextual. Understanding the phenomenon, its spheres, and dimensions provides health professionals with a basis and resources to act as facilitators and promoters of hope, contributing to construct meaning and significance and to strengthen internal resources that support coping for both children and adolescents and their families (6).

In this scenario, a scoping review is configured as an appropriate methodological approach to broadly and systematically map how the meanings, perceptions, and manifestations of hope have been described in the literature and how they have contributed to coping by children and their families in the context of childhood cancer. This strategy enables identifying and analyzing conceptual and empirical gaps which can guide and direct future research, supporting clinical practices that are more sensitive to the subjective processes involved.

A preliminary search of the International Prospective Register of Systematic Reviews (PROSPERO), JBI Evidence Synthesis, and The Cochrane Database of Systematic Reviews was conducted in the first half of 2024, and no previous reviews with a similar focus were identified. This study is justified given the lack of published or ongoing systematic and scoping reviews on this topic, considering that mapping the evidence of this phenomenon is essential for identifying gaps which can contribute to the work of healthcare professionals as a network that facilitates and promotes hope.

This study aimed to map scientific evidence on the hope of children, adolescents, and their families, as well as the meanings, perceptions, and manifestations of hope associated with coping during childhood cancer treatment.

## Methods

This study consists of a scoping review structured according to the five steps established by the Joanna Briggs Institute (JBI), namely: defining the research question, identifying relevant studies, selecting and including studies, organizing data, and finally, grouping, synthesizing, and reporting results (7).

The research protocol was registered and updated in the Open Science Framework (OSF) on October 7, 2024.

The research question and article eligibility criteria utilize the PCC (Population, Concept, and Context) approach.

- P (Population): families of children and adolescents up to 19 years old undergoing cancer treatment, including themselves and their siblings;
- C (Concept): experiences of hope and manifestations of hope, including concepts, meanings, and perceptions;
- C (Context): cancer treatment.

The concept of hope described by Dufault and Martocchio (5) was adopted, according to which:

Hope is a multidimensional dynamic life force characterized by a confident yet uncertain expectation of achieving a future good which, to the hoping person, is realistically possible and personally significant (5, p. 380).

Thus, the following research question was outlined: what are the meanings, perceptions, and manifestations of hope associated with coping for children, adolescents, and their families during childhood cancer treatment?

The databases and search strategies were selected after conducting pre-tests, carried out through several exploratory searches with the support of a librarian specializing in research. The final search strategy integrated controlled Medical Subject Heading descriptors (MeSH), combined using the Boolean operators AND and OR. The complete strategy is available in Supplementary Table S1.

The search was conducted in the following databases: Cumulative Index to Nursing and Allied Health Literature (CINAHL), National Library of Medicine (NIH PubMed), Scientific Electronic Library Online (SciELO), American Psychological Association (APA PsycINFO), SCOPUS, and LILACS on May 19, 2025, with an update on February 16, 2026. These databases were selected because they bring together the most relevant scientific production on hope in the pediatric oncology context, comprising multidisciplinary literature essential to understanding the psychological, social, and clinical aspects of the phenomenon.

Articles with accessible full text were included, such as original studies, randomized and non-randomized clinical trials, bibliographic, documentary, experimental, field, and case studies, as well as review studies and reflective or theoretical productions. Different methodological approaches were considered, including quantitative, qualitative, theoretical research, or mixed methods studies, primary and secondary studies, and data triangulation. No restrictions were applied regarding language or publication period.

All publications that included: children and adolescents diagnosed with cancer over the age of 19; studies which addressed the experience of formal caregivers or those who were not parental or family caregivers; investigations focused on the perception of health professionals; studies whose main focus was the patient's experience in relation to the cancer diagnosis; studies which examined parental or family responses to new treatment technologies; studies focused on the acceptance or not of genetic testing or counselling; studies oriented towards the analysis of the biological responses of the disease or the treatment implemented; studies referring to other serious childhood illnesses; studies focusing on the quality of life of parental caregivers; research protocols; and studies whose objective was to understand parents' perception of support groups were all excluded.

The mapping of scientific literature indexed in the selected databases was chosen in order to ensure access and validated methodological rigor. No initial filters were applied to exclude specific types of documents; however, given the breadth of the construct, the searches were limited to the title and abstract fields to ensure relevance and conceptual viability. The final sample prioritized journal articles and reviews considering the depth and complexity of the concept analysis, as well as retrieval unavailability in other formats identified in the initial screening. Studies unavailable for retrieval, including dissertations that did not have digital support or public access, were excluded after exhaustive searches. Abstracts, letters to the editor, and conference proceedings were also disregarded.

All identified studies were imported into the Rayyan reference management software (https://www.rayyan.ai/), where duplicates were removed. The selection of articles occurred in two stages: initial screening by reading the title and abstract, followed by evaluation of the full text. This process was conducted based on the inclusion and exclusion criteria defined in the review protocol. The references of the selected articles were systematically analyzed in search of additional literature relevant to this review (8).

The screening and selection of the identified publications were conducted independently using Rayyan by three designated reviewers from the research team (First, third and fourth authors). Discrepancies and disagreements regarding the inclusion or exclusion of any individual study, at either the title/abstract screening stage or the full-text evaluation stage, were resolved through discussion and consensus among the three reviewers, with a fourth independent reviewer (second author) consulted to mediate when consensus could not initially be reached.

Data extraction from evidence sources followed the instrument recommended by the Joanna Briggs Institute (JBI template source of evidence details, characteristics, and results extraction instrument) for detailing the characteristics of the studies and relevant findings. The following information was collected from the studies: author, year of publication, country where the study was conducted, publication database, objective, participants/sample, study setting, methodology, main results related to evidence of hope in the oncological context, and identified implications (7).

The analytical process included both characterization of the studies and publication trends, as well as a synthesis of evidence related to the hope of children and adolescents and their families during cancer treatment. The evidence of hope was fully extracted and organized into thematic categories that encompass meanings, perceptions, and manifestations of hope, as well as factors which promote and threaten hope, in accordance with the theoretical frameworks that underpin this scoping review. Additionally, an analysis of the implications presented in the studies was conducted with the aim of identifying gaps and challenges for practice and research regarding hope and coping in the context of pediatric oncology (7, 9).

## Results

The search retrieved 4,380 studies; 1,782 were excluded due to duplication, and 2,598 had their titles and abstracts read. At this stage, 2,486 were excluded, leaving a total of 112 studies for full-text reading. Of these, 29 were unavailable either due to institutional access or the need for a paid subscription, and 83 were read in full text. Then, 52 of the 83 studies were excluded for not meeting the established eligibility criteria (50 did not focus on the parental or family experience of hope in coping; 1 sought to evaluate parents' responses regarding a support group; and 1 evaluated caregivers’ responses regarding the diagnostic moment, specifically). Thus, the final sample consisted of 31 studies.

The review was reported based on the Preferred Reporting Items for Systematic Reviews and Meta-Analyses extension for Scoping Reviews (PRISMA-ScR) checklist to ensure methodological integrity and transparency of the process, supplemented by the PRISMA flowchart for the study selection process (Figure 1) (10).

**Figure 1:**
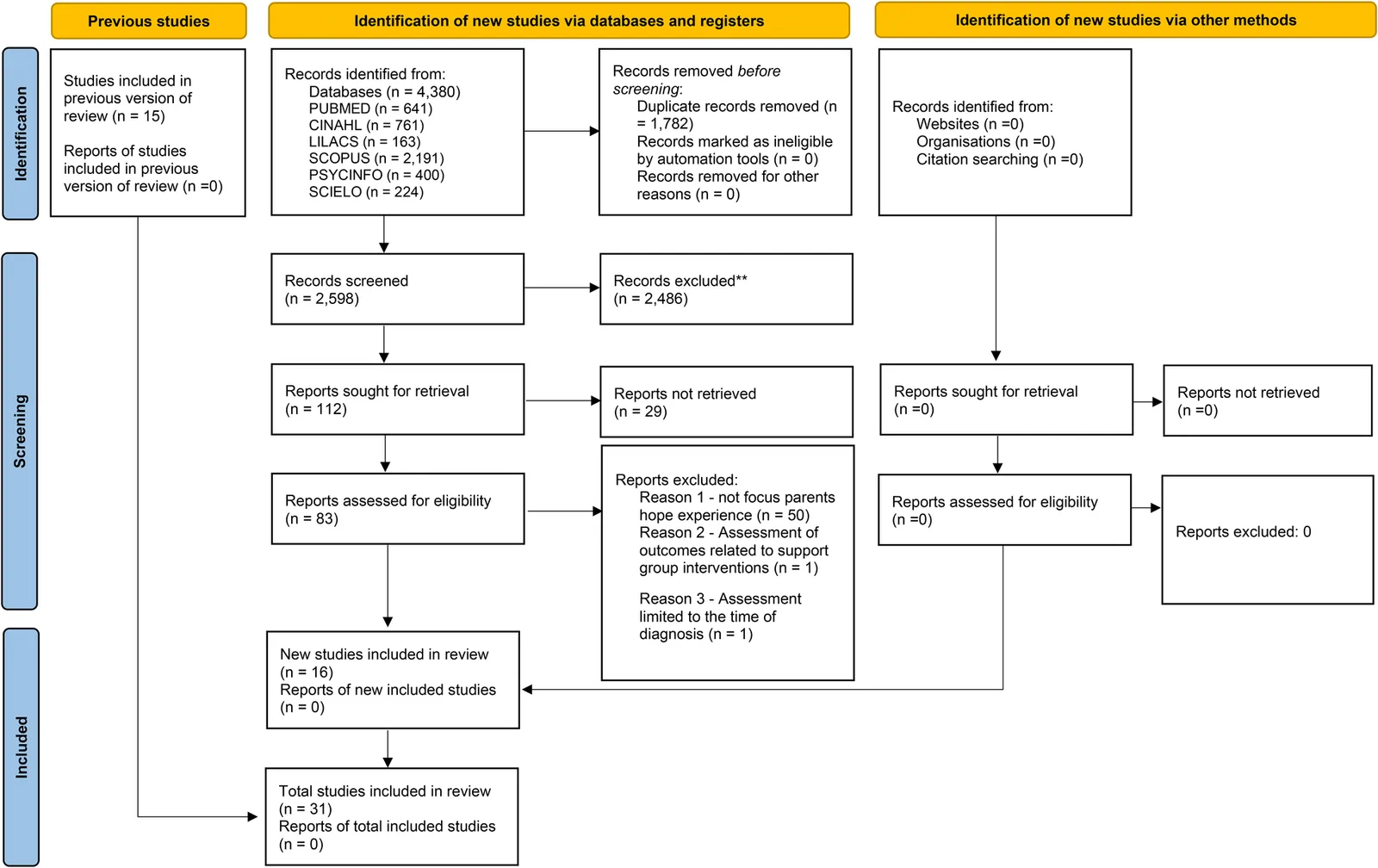
PRISMA flowchart for study selection.

Characterization of the studies is presented in Table 1, including study identification, country, publication date, objective, participants, and study design. The studies were identified with the letter “S” in reference to the study followed by a numeral, such as “S1” for study 1. Regarding the year of publication, there was a growth in interest in the topic in the last 10 years (*n* = 20), with publications written in English predominating (90%). The geographical production for the country of publication is diverse, encompassing 19 countries, with emphasis on the United States of America (USA) (*n* = 8) and Israel (*n* = 3). Regarding the method and design, studies which used qualitative approaches through face-to-face interviews (*n* = 16) or telephone interviews (*n* = 1) prevailed, followed by quantitative studies that use psychometric scales to measure the construct (*n* = 9), systematic and scoping reviews (*n* = 4), and mixed-methods studies (*n* = 1).

**Table 1:** Characterization of the studies included in this review.

Identification—ID	Country/Year of publication	Objective	Participants	Design
S1 (11)	Iran/2025	Investigate the relationship between the needs for supportive care, resilience, and hope in mothers of children with cancer.	200 mothers of children (<18 years) undergoing cancer treatment	Cross-sectional correlational study (quantitative approach)
S2 (12)	China/2025	Examine the feasibility and preliminary effectiveness of a narrative-based card game intervention and hope techniques (HT-NCG).	60 pediatric patients (12–17 years) with cancer	Randomized controlled trial (RCT) (quantitative approach)
S3 (13)	France/2024	Explore how both partners experience relationship dynamics and hope within the couple.	7 couples	Study with semi-structured interviews and interpretive phenomenologic al analysis (qualitative approach)
S4 (14)	Brazil/2024	Analyze the effectiveness of family interventions in coping and enhancing problem-solving skills in parents and/or caregivers of children and adolescents during and after cancer treatment.	Not applicable (45 studies)	Scoping review
S5 (15)	Israel/2024	Explore the role of perceptions of the impact of the disease in mediating the link between hope, social support, and subjective well-being in parents of children with cancer.	108 parents of children with cancer (mothers: 70.4%)	Cross-sectional study with questionnaire application and statistical analyses (quantitative approach)
S6 (16)	Turkey/2024	Examine the effects of psychological resilience, spiritual well-being, and some child- and parent-related factors on parents’ experience of hope while their children are undergoing cancer treatment.	120 parents of children with cancer (mothers: 57.5%)	Cross-sectional correlational study (mixed-methods approach)
S7 (17)	Israel/2024	Identify profiles based on two distinct resources: sense of hope (personal resource) and perceived social support (social resource).	111 adolescent siblings of children with cancer	Quantitative approach using self-report questionnaires
S8 (18)	Malaysia/2023	Explore spirituality among Malaysian Muslim caregivers of children with acute lymphoblastic leukemia.	13 parental caregivers of children and adolescents with cancer	Phenomenologic al study (qualitative approach)
S9 (19)	Japan/2023	Synthesize the qualitative experiences of children with cancer, parents, and healthcare professionals, as well as their social interactions during the end-of-life decision-making process in the context of pediatric oncology.	Not applicable (21 studies)	Systematic review and meta-synthesis
S10 (20)	United Kingdom/2022	Explore the values and preferences of parents that underpin decision-making regarding the treatment of children receiving cancer-targeted therapy for a poor-prognosis cancer.	Not applicable (12 studies)	Scoping review
S11 (21)	USA/2022	To increase healthcare teams’ understanding of the end-of-life care preferences of bereaved parents through narratives.	5 parents of children with cancer (3 women and 2 men)	Qualitative study with coding analysis
S12 (22)	USA/2021	To explore parents’ perspectives to begin to understand communication.	80 parents of children with cancer (mothers: 67)	Qualitative study with semantic content analysis
S13 (23)	Iran/2021	To examine the relationship between the level of hope in mothers who have a child with cancer and its related factors in Iran.	240 mothers	Cross-sectional correlational study with a quantitative approach
S14 (24)	USA/2020	To examine the evidence on the value of spirituality/spiritual care in minimizing the vulnerability of parents whose children have been diagnosed with cancer and who have faced end-of-life care.	Not applicable (14 studies)	Scoping review
S15 (25)	Ghana/2020	To explore the psychological and spiritual well-being of family caregivers of children with cancer in a teaching hospital in Ghana.	13 (12 mothers and 1 father)	Exploratory descriptive study with a qualitative approach and thematic content analysis
S16 (26)	Colombia/2020	To understand the feelings of children and adolescents diagnosed with cancer through research on the changes they experience.	Children and adolescents diagnosed with cancer	Phenomenologic al qualitative study
S17 (27)	Indonesia/2018	To explore the experiences of parents caring for their children with cancer under palliative care in depth, focusing on emotional and spiritual aspects.	10 parents (8 mothers and 2 fathers)	Descriptive phenomenologic al qualitative study
S18 (28)	Argentina/2017	To explore how parents of children with cancer construct treatment narratives by including events that did not happen but could have, so-called “unnarrated”, and how these elements reveal their fears, hopes, and desires.	17 parents (13 mothers and 4 fathers)	Qualitative study with narrative interviews
S19 (29)	USA/2018	To investigate the sources of hope for parents of children with cancer during the first year of treatment, considering different prognoses.	374 parents	Prospective and longitudinal cohort study based on questionnaires with a quantitative approach
S20 (30)	USA/2017	To understand how parents define hope and how healthcare professionals influence that hope.	50 parents (48 mothers and 2 fathers)	Qualitative study
S21 (31)	USA/2015	To explore the meaning of hope for parents of children with advanced cancer and how it coexists with knowledge of the prognosis.	Parents of 32 children and adolescents with cancer	Qualitative study
S22 (32)	Portugal/2015	To analyze associations between the meaning of family rituals and quality of life in children with cancer and their parents, considering family cohesion and hope as mediators.	389 dyads (children and adolescents with cancer and one parent)	Quantitative study
S23 (33)	Canada/2013	To explore the experience of hope for parents when their children have cancer with a poor prognosis and to identify facilitators and barriers to maintaining hope in this context.	35 parents (26 mothers and 9 fathers)	Qualitative study based on grounded theory
S24 (34)	Israel/2013	To prospectively and longitudinally understand how parental hope evolves during the first year after the diagnosis of difficult-to-treat cancer in their children.	35 parents (26 mothers and 9 fathers)	Qualitative study based on grounded theory
S25 (35)	Colombia/2013	To describe the concept of hope that parents of children between 0 and 15 years of age diagnosed with cancer have.	11 parents (7 women and 4 men)	Interpretive qualitative study, descriptive-exploratory type
S26 (36)	Taiwan/2009	Describe the essence of coping experiences of Taiwanese adolescents with cancer.	10 adolescents	Qualitative study approach
S27 (37)	Australia/2008	Explore the experiences of families when a child with cancer relapses, focusing on uncertainty, hope, and fear.	12 families	Qualitative study approach
S28 (38)	USA/2007	Discuss the role of hope in prognostic communication in pediatric oncology.	194 parents (mothers: 153)	Quantitative study approach
S29 (39)	Finland/2007	Describe hope in parents of adolescents with cancer and the factors which influence it.	9 parents (5 mothers and 4 fathers)	Qualitative study approach with inductive content analysis
S30 (40)	Portugal/2001	Describe the structure of a hopeful relationship.	6 parents (4 women and 2 men)	Qualitative study approach, interpretative phenomenologic al analysis
S31 (41)	USA/2000	To develop and apply an appropriate quantitative threat appraisal measure for children with cancer and to investigate how different dimensions of threat relate to indicators of psychosocial adjustment, including hope.	40 children and their parents	Cross-sectional study with quantitative approach

The included studies reveal great diversity in terms of participants. Although maternal participation predominates, participation of children, adolescents, siblings, and parents was also recorded and analyzed. The average age of children and adolescents with cancer was 9.46, and 15.18 for siblings. Mothers and fathers were classified as young adults, with no numerical data presented in most studies.

Regarding the type of cancer, leukemias, lymphomas, and central nervous system tumors predominated, followed by other solid tumors. Parental education was described variably. Between 23% and 35% had secondary education or below; 40%–64% had higher education; and 14%–24% had postgraduate studies. The marital status of married or living in a stable union predominated. The rate of parents who were married or living as married in large samples, such as in multicenter studies, was 82%.

Regarding family composition, the number of children ranged from 2 to 4 per family in the studies that reported it. Economic variables were not frequently detailed, but there are records indicating income lower than family expenses.

The evidence based on the data extraction was then grouped into thematic categories that reflect the complexity of the construct, as shown in Table 2.

**Table 2:** Evidence of hope.

Thematic categories	Evidence
Meanings of hope Cognitive Dimension (*Thought processes, symbols and meanings*)	An internal resource of motivational state, positive beliefs, and vital force to keep fighting, even amidst what seems impossible [S3, S4, S10, S24, S26, S29, S30].
	Energy. The sap of life and a ray of light in the darkness [S6, S27].
	Hope for healing, miracles, recovery, and survival [S10, S15, S16, S17, S21, S27].
	Reinterpretation of the hope for healing as hope for relief from suffering and a peaceful death [S9, S11, S17].
	“When the hope of no suffering surpasses the hope of being with one’s child, a transformation of hope occurs.” [S9].
	Acceptance of God’s will and faith in God’s provision [S8, S20].
	Hope is a mixture of the fear of losing the child and the faith that everything will be alright [S25].
	Hope as guidance for life and the future [S23, S29].
	Beyond a prognostic calculation, but anchored in reality through information, through perceived truth [S30].
	Feeling of “I can” in the face of suffering [S30, S31].
Perceptions of hope Affective Dimension (*Emotions and associated feelings*)	Motivating source and essential pillar of struggle and coping to overcome pain and suffering [S3, S4, S25].
	They feel that hope arises when surrendering the situation to God (who sustains them), coexisting with fear and lack of control [S8, S17, S25].
	Unstable experience of fluctuating between hope (flashes of light) and fear (darkness) [S6, S9, S10, S24, S27].
	They value professionals capable of sustaining hope and realism (without false expectations). A “roadmap” with possibilities for action and continuation of care [S11, S12].
	They perceive the coexistence of realistic and impossible goals, but trusting in a greater meaning [S21, S30].
Manifestations of hope Behavioral Dimension (*Actions to sustain hope*)	Future projections, redefining goals, positive attitudes involving an optimistic perspective [S1, S2, S5, S11, S15, S24, S25].
	Living “day to day” and maintaining normalcy as much as possible [S11, S21, S24, S29, S30].
	Proactive search for knowledge and realistic medical information [S28, S30].
	Constant spirituality: Prayer, fasting, rituals, and surrender to God [S6, S8, S15, S17, S23, S25].
	Active participation in treatment and care, seeking other therapies and treatments, survivor stories [S1, S2, S6, S9, S10, S12, S19, S27]
Factors which promote hope Affiliative Dimension (*Social relations and transcendence*)	Communication and Relationship with Healthcare Professionals: Honest dialogue, detailed disclosure of the prognosis, active listening, sensitivity, clarity, and being treated as a person [S4, S9, S10, S11, S12, S19, S20, S21, S26, S28, S29, S30].
	Support: Couple unity, family rituals, family network, parent groups, positive reports, friends, and supportive presence of the team [S3, S4, S5, S6, S7, S12, S13, S22, S25, S26, S29, S31].
	Spirituality: Faith, prayer, and religious support [S3, S4, S6, S8, S10, S13, S14, S15, S17, S23, S25, S29].
	Positive responses to treatment [S23, S24, S27].
	Individual factors: Higher maternal education level, better economic status, constructive adolescent personality, mood, child’s own resilience, presence of pets and structured play [S1, S2, S6, S24, S25, S29].
Factors threatening hope Contextual and Temporal Dimensions (*Uncertainties and circumstances*)	Clinical Events: Relapse, uncertainty of prognosis, physical pain, severe side effects, real perceptions of the impact of the disease, child’s suffering, and proximity to death [S1, S2, S5, S6, S9, S10, S15, S16, S17, S20, S21, S23, S24, S26, S27, S28, S29, S31].
	Care Failures: Objectification (being treated as an object), lack of information, lack of realistic honesty, false hope, one-off and late conversations, conflicting messages, abrupt communication without preparation, divergent information among the team, feeling of loss of control, and silencing of fears [S11, S12, S17, S20, S21, S23, S29, S30].
	Psychosocial Context: Economic instability, unemployment, social isolation, abandonment by peers and “hopeless truth” (cruel realism), attempts to reduce the other’s hope in the perception of an impossibility of hope (couple) [S1, S2, S3, S6, S7, S8, S15, S18, S22, S24, S25, S29, S30].
	Internal Threats: Loss of control/autonomy, emotional exhaustion of parents, perception of injustice and threat to identity “This makes me a worse/different/rejected person” [S18, S23, S24, S26, S29, S31].

Hope, in the context of pediatric oncology, represents a key element in coping with childhood cancer and preserving emotional integrity, which connects to the construction of hope understood by Dufault and Martocchio as a vital and dynamic force. In the analysis of the studies, hope is signified as a “ray of light” which sustains the child, the adolescent, and their family in the face of so much uncertainty. As a coping resource, hope gives meaning to the experience of becoming ill with cancer and promotes resilience. Its manifestations permeate the generalized sphere, with a focus directed towards a broad sense of the future, and also the particularized hope more focused on concrete goals such as curing the disease and treatment success.

Constant transformations occur throughout the course of cancer given the dynamic nature of hope, accompanied by fluctuations in clinical status and prognostic uncertainties. The cure, a great expectation in the face of a sudden and uncertain diagnosis, can be given new meaning by the search for peace found in the relief of symptoms, comfort, or even in a dignified death. These transformations can be influenced by the affective dimension, with flashes of light and darkness coexisting, but also by the cognitive dimension, in which the search for information and truth sustains realistic hope. There is always hope (5).

## Discussion

Hope is not a static trait, but a complex, active, and dynamic process composed of two spheres: the generalized one, which provides an “umbrella” promoting motivation and protection against suffering and despair; and the particular one, taking the reins and directing the trajectory against cancer towards specific, real, and meaningful goals. Hope can be cultivated and nurtured, acting in the present and striving to direct the future (5).

The understanding of hope as a vital and multidimensional force remains consistent across various circumstances of childhood illness. The evidence reported in this review corroborates the literature by showing that, regardless of the context (whether a chronic condition or a complex Hematopoietic Stem Cell Transplantation process), hope reveals itself as a resource for relief and strengthening (42, 43), sustaining emotional integrity and boosting resilience even in the face of so much uncertainty and high levels of psychological suffering. Hope, in its metamorphic nature, functions as a “lifeblood” (S6, S27).

The findings of this review indicate that hope is initially strongly directed toward the expectation of a cure or the success of treatment, which gives the future a positive perspective aligned with the sense of present and future of the Temporal Dimension (S2, S10, S15, S21). However, the qualitative evidence reveals a dynamic transformation process. A re-signification process is observed in the face of a worsening prognosis, disease progression, or the approach of the end of life, and the hope for a cure is remodeled by the hope for relief from suffering, symptoms, hope for comfort, and for a dignified and peaceful death (S9, S11, S17). This natural movement of feelings confirms what is proposed by the theory in the Affective and Contextual Dimension, namely that there is always hope, even if the objective changes to preserve the meaning of life until the end (5, 44).

This redirection and persistence of hope at the end of life closely aligns with the foundational work of Elisabeth Kübler-Ross (45), a seminal teacher of hope in the study of dying. Kübler-Ross (45) observed that even in the deepest stages of terminal illness, hope remains a persistent, vital, and protective force, transforming from an expectation of cure to a focus on pain relief, comfortable moments, and peaceful closure, reinforcing that hoping is an essential mechanism for coping with death and life-threatening conditions. Reframing requires cognitive adjustments to mitigate impairment in family functioning and preserve the parents' ability to provide care. The goal is transformed into living deeply, creating memories, protecting the child's identity, and seeking concrete goals to provide comfort and peace (46).

Relationships that extend beyond oneself refer to the Affiliative Dimension, based on interpersonal bonds, meaningful social interactions, spirituality, and connection with professionals working in the care network for children and adolescents with cancer and their families (5). In this review, the relationship between the health team and the family emerges as a determinant directly related to realistic hope.

The actions of health professionals are identified as an important resource for promoting hope (S1, S12, S20, S28, S30). Their actions not only reflect technical care, but also a source of information that promotes the so-called “scanning of reality”. This concept, described as a mental process of the Cognitive Dimension, connects with the Affiliative Dimension, as the nurse acts as a facilitator when supporting the family in understanding what is realistically possible, bringing confidence and support to hope (5).

Nurses working with children with chronic conditions recognize different manifestations of hope related to the child's clinical condition and use strategies to cultivate it, such as therapeutic presence, active listening, empathetic communication, offering clear information, valuing progress in treatment, spiritual support, and encouraging the construction of positive future perspectives (47). Nurses identify that the quality of the relationship with the family, institutional resources, and the approach of the health team influence hope cultivation. These findings suggest that the therapeutic use of hope, combined with structured assessments and individualized interventions, can contribute to better outcomes in the care of children with chronic diseases and their families (47).

Truth and honesty are valued. Studies show that hope is not sustained by false expectations, but by honest, detailed, and sensitive dialogue (S20, S28, S30). When communication is accessible, it enables the family to develop trust and security in planning for the future, even if uncertainty or threatening prognoses persist. These actions can be developed through playful tools that simplify the narrative (goals and future paths) and practically support hope (S2).

In this communication process, the ethical dilemma of the “white lie” (benevolent deception or information filtering) represents a prominent clinical reality. Healthcare professionals and parents often resort to “white lies” out of a protective instinct, hoping to shield the child and family from the devastating impact of the end of life and keep hope alive (S2, S11). However, as evidenced in the literature, although a harsh and direct reality can be traumatic, complete reliance on “white lies” can undermine the patient's trust, silence their implicit fears, and hinder essential conversations about goals of care and quality of life (S20, S28, S30). Therefore, rather than outright deception, clinical practice should move toward a balanced approach to “sensitive truth-telling,” in which information is shared progressively and gently, preserving the patient's dignity and supporting hope for a comfortable and meaningful present.

In contrast, communication failures such as discrepancies in information among the team, words or attitudes that silence fears and anxieties, or frequent harsh and cruel dialogue which presents the truth without any room for hope are serious and devastating threats that produce estrangement, suffering, and emotional exhaustion (S11, S12, S17, S20, S21, S23, S29).

The performance of the health team and the social support received by the family are central elements and significant predictors of psychological stress and caregiver burden. These elements exert influences capable of fostering or threatening a sense of hope. Constructing therapeutic alliances based on trust and honesty helps the family to make difficult but conscious decisions (48).

Focusing on action orientation, the Behavioral Dimension encompasses movements in pursuit of achieving hope and sustaining vital forces for daily coping (5). This dimension was primarily identified in this review in pursuit of balancing treatment and family dynamics.

Living “day-to-day” and preserving as much normalcy as possible are the behavioral expressions most present in the studies, both in hospital settings and at home (S11, S21, S24, S29, S30). As described by the theory, hope acts as a “boost” which encourages and motivates the person to face daily demands, even when fatigue is constantly present. These actions prevent stagnation and self-pity. The presence of natural dynamics and family rituals allows the family to use hope as a form of relief and mitigation against the treatment intensity (5).

The proactive search for information and involvement in care are also manifestations of acting in their parental role (S28, S30). This behavior, described in the Behavioral Dimension, contributes to reducing feelings of powerlessness and helplessness, while strengthening confidence in one's own ability to positively influence the child's well-being (5).

Spirituality appears in this review in expressive form and frequency as a fundamental active practice and not just a feeling. In Dufault and Martocchio's theory (5), religious actions are part of the Behavioral Dimension when related to the fulfillment of a hope (5). Religious rituals represent legitimate strategies for families (S6, S7, S15, S23, S25). Trust in God or a higher power seeks to sustain faith in achieving what seems impossible, providing meaning and resilience so that the family can accept what cannot be changed without losing heart in continuing with the treatment (S8, S20).

Faith and spirituality are essential coping mechanisms which act as protective mechanisms capable of mitigating anxiety and sadness by alleviating the suffering of experiencing cancer or finitude (42, 44). Upholding the right to “hope” strengthens and validates family unity by supporting its resilience and functioning. These actions can mediate the control and balance between suffering and psychological well-being (44).

The cultural and socioeconomic implications involving hope are underexplored. Most of the included studies present evidence from developed countries. The results presented herein confirm that factors such as economic status, unemployment, and favorable income have a direct impact on caregiver burden, which consequently can affect maintaining hope. Geographic and social disparities reinforce the need to consider vulnerabilities and ethnic minorities in order to ensure promoting hope in the most diverse contexts (S30) (46, 48).

Current scientific research recognizes the importance of the diverse interactions of social support; however, the analysis of studies reveals fragmentation, focusing on understanding individual experiences of hope, especially maternal ones, within the family context, and not as a broader dynamic unit. Future studies could seek to capture the perspectives and experiences of all members who compose the child's and adolescent's care network, whether siblings or other significant and active people in family life, regardless of blood ties.

A longitudinal approach which follows the transformation of family hope as a broader unit at different points in the cancer treatment trajectory could enable deeper understanding of hope as a family process and interactional construct.

This review rigorously followed the recommendations of the Joanna Briggs Institute (JBI); however, it is important to consider some limitations. The search prioritized indexed literature to ensure methodological rigor. However, grey literature, such as theses and dissertations, selected after reading the titles and abstracts, were unavailable for full-text retrieval. A total of 29 studies that qualified after the initial screening were unavailable for full-text reading.

## Implications for practice

Understanding the hope of children, adolescents, and families dealing with cancer as a multidimensional process is challenging, but it provides healthcare professionals with resources for actions and attitudes that foster hope. Recognizing what promotes and threatens hope influences trust and development of meaningful relationships.

Providing space for the expression of emotions, validation of feelings, and uncertainties are practical strategies which demonstrate empathy and support the family in processing and reframing these feelings.

Maintaining a clear, timely, and informative dialogue can facilitate acceptance of situations where reality cannot be changed, but hope can still be held. Supporting the family's role in practical and daily care can increase the sense of control and help reduce feelings of inadequacy. Promoting a safe environment that encourages reflection on values, meanings, and the purpose of life, especially when the hope of a cure needs to be reframed, can transform experiences.

## Implications for research

This review acknowledges gaps and the need for research that broadens family representation. Most studies focus on capturing the experiences of isolated members, such as mothers or siblings, for example (S7, S13). It is essential to consider the perspective of people, whether family members or not, who actually live, participate in, and are significant in the life and care of the child and adolescent with cancer.

Understanding the family's experience of hope as a unit of support and care and its repercussions on family functioning is insufficient. This in-depth knowledge enables better analysis of the movement and transformation of hope as a family process and interactional construct. This integration is essential because childhood cancer affects the family intensely and overall, requiring readjustments and coping strategies in all dimensions of its functioning. Understanding hope in pediatric oncology remains an open field for future research willing to expand parental and individual roles. Furthermore, it is recommended to deepen the understanding of hope with temporal snapshots of the experiences of illness due to cancer in children and families.

The predominance of publications from the United States of America and in English is another point to consider. This geographical barrier points to the need to represent family diversity in the cultural and socioeconomic contexts of hope, its nuances and variations, in a broader and fuller way. A total of 19 countries are represented in this review; however, most of them contribute only one study.

## Conclusion

This scoping review mapped and systematized the evidence on family hope in the context of pediatric oncology and confirmed its presence as a vital, dynamic, and multidimensional force, fundamental to coping with the disease for both the child and adolescent, and for their family. Hope was present after the sudden diagnosis through the intense desire for a cure; during the uncertain treatment, but which persisted in the search for a positive future; and in the face of the end of life, seeking comfort, dignity, and peace. Its metamorphic nature sustained the strength and faith in all the complex oscillations of the trajectory of coping with cancer.

The relationship built with healthcare professionals promotes interactions which directly impact the family's hope, shaping feelings and behaviors. The child, the adolescent, and their families find a source for connection in these professionals to a tangible reality and support of their hope. However, the lack of information, sensitivity, and support can devastate any spark of hope. The care provided by these professionals must transcend clinical practice, including support for beliefs and encouragement to maintain the normality of family dynamics, providing protection for emotional integrity.

Regardless of prognostic uncertainty, hope remains active and alive in all its multidimensional complexity. This recognition is essential to care which seeks to support autonomy and provide comfort and security to families, children, and adolescents experiencing cancer. To operationalize this support during the end-of-life phase, healthcare teams must transition from curative efforts to an active and structured approach to continuous support. In this phase, hope is maintained by carefully preserving a sense of “living as normally as possible.” Clinically, this requires maintaining active medical monitoring, symptom-control protocols, or supportive interventions such as metronomic therapy, which can provide disease stabilization with low toxicity while preserving the child's daily routine. Such a practical clinical approach prevents the devastating feeling of abandonment for both the patient and the family, reassuring them that active and dedicated care will continue and that they will be closely accompanied and supported until the very end.

## Data Availability

The original contributions presented in the study are included in the article/Supplementary Material, further inquiries can be directed to the corresponding author.

## References

[1] Sultan I Alfaar AS Sultan Y Salman Z Qaddoumi I. Trends in childhood cancer: incidence and survival analysis over 45 years of SEER data. PLoS One. (2025) 20(1):e0314592. 10.1371/journal.pone.0314592 39752445 PMC11698462

[2] Beltrami A Hilliard A Green AL. Demographic and socioeconomic disparities in pediatric cancer in the United States: current knowledge, deepening understanding, and expanding intervention. Cancer Epidemiol. (2022) 76:102082. 10.1016/j.canep.2021.102082 34923289

[3] Souza RLA Mutti CF Santos RP Oliveira DC Okido ACC Jantsch LB. Hospitalization perceived by children and adolescents undergoing cancer treatment. Rev Gaucha Enferm. (2021) 42:e20200122. 10.1590/1983-1447.2021.20200122 34037109

[4] Vieira AC Cunha MLR. My role and responsibility: mothers’ perspectives on overload in caring for children with cancer. Rev Esc Enferm USP. (2020) 54:e03540. 10.1590/S1980-220X2018034603540 32187307

[5] Dufault K Martocchio BC. Hope: its spheres and dimensions. Nurs Clin North Am. (1985) 20(2):379–91. 10.1016/S0029-6465(22)00328-0 3846980

[6] Silveira AO Wernet M Franco LF Dias PLM Charepe Z. Parents’ hope in perinatal and neonatal palliative care: a scoping review. BMC Palliat Care. (2023) 22:202. 10.1186/s12904-023-01324-z 38110974 PMC10726497

[7] Aromataris E Lockwood C Porritt K Pilla B Jordan Z. JBI Manual for Evidence Synthesis. JBI (2024).

[8] Ouzzani M Hammady H Fedorowicz Z Elmagarmid A. Rayyan—a web and mobile app for systematic reviews. Syst Rev. (2016) 5:210. 10.1186/s13643-016-0384-4 27919275 PMC5139140

[9] Munn Z Aromataris E Tufanaru C Stern C Porritt K Farrow J. The development of software to support multiple systematic review types: the Joanna Briggs institute system for the unified management, assessment and review of information (JBI SUMARI). Int J Evid Based Healthc. (2019) 17(1):36–43. 10.1097/XEB.0000000000000152 30239357

[10] Tricco AC Lillie E Zarin W O'Brien KK Colquhoun H Levac D. PRISMA extension for scoping reviews (PRISMA-ScR): checklist and explanation. Ann Intern Med. (2018) 169(7):467–73. 10.7326/M18-0850 30178033

[11] Alizadeh-Khanghahi M Farshbaf-Khalili A Alizadeh M Jabraeili M. How supportive care needs influence resilience and hope in mothers of children with cancer? BMC Pediatr. (2025) 25:743. 10.1186/s12887-025-06145-y 41039392 PMC12492697

[12] Liu Q Ho KY Lam KKW Wong FKY Lam W Cheng F. Preliminary effectiveness and feasibility of an integrated hope techniques and narrative-based card game intervention for pediatric cancer patients in China: a randomized controlled trial. BMC Med. (2025) 23:449. 10.1186/s12916-025-04287-5 40745312 PMC12315444

[13] Milville L Lelorain S Antoine P. Hope and relationship dynamics among couples faced with their child’s cancer: a dyadic phenomenological interpretative analysis. Eur J Oncol Nurs. (2024) 72:102690. 10.1016/j.ejon.2024.102690 39303329

[14] Prates PEG Correa-Júnior AJS Russo TMS Paraizo-Horvath CMS Teles AAS Sonobe HM. Effectiveness of family coping interventions in improving problem-solving skills in the care of children and adolescent cancer survivors during and after treatment: a scoping review. Nurs Rep. (2024) 14(3):2153–78. 10.3390/nursrep14030161 39311170 PMC11417838

[15] Schwartz-Attias I Krulik T Ronen T. Well-being in parents of children with cancer: illness perceptions’ mediating role for hope and social support. Front Psychol. (2024) 15:1206520. 10.3389/fpsyg.2024.1206520 39021645 PMC11252627

[16] Ozdemir Koyu H Algül G Altay N Kilicarslan E. The effect of psychological resilience and spiritual well-being on the experience of hope in parents of children with cancer. J Pediatr Nurs. (2024) 76:e34–41. 10.1016/j.pedn.2024.01.016 38307757

[17] Hamama L Levy S. Adolescent siblings of children with cancer: resource-based profiles, normalization, and search for meaning in life. J Adolesc. (2024) 96(2):221–34. 10.1002/jad.12269 37926934

[18] Chong ASS Ahmad MB Alias H Hussain RBI Lateh AB Chan CMH. Spiritual coping among families of children with cancer: a qualitative study. Asia Pac J Public Health. (2023) 35(6–7):408–12. 10.1177/10105395231190830 37515530

[19] Hirata M Kobayashi K. Experiences with the end-of-life decision-making process in children with cancer, their parents, and healthcare professionals: a systematic review and meta-ethnography. J Pediatr Nurs. (2023) 69:e45–64. 10.1016/j.pedn.2022.12.004 36586777

[20] Pearson H Bryan G Kayum C Gibson F Darlington AS. Parent values and preferences underpinning treatment decision-making in poor-prognosis childhood cancer: a scoping review. BMC Pediatr. (2022) 22:595. 10.1186/s12887-022-03635-1 36229792 PMC9563461

[21] Robert R Razvi S Triche LL Bruera E Moody KM. Bereaved parent perspectives on end-of-life conversations in pediatric oncology. Children. (2022) 9(2):274. 10.3390/children9020274 35204993 PMC8870516

[22] Sisk BA Schulz GL Blazin LJ Baker JN Mack JW DuBois JM. Parental views on communication between children and clinicians in pediatric oncology: a qualitative study. Support Care Cancer. (2021) 29(9):4957–68. 10.1007/s00520-021-06047-6 33569673 PMC8295182

[23] Fathollah Zadeh E Parry Y Eshghi P. Hope in Iranian mothers of children with cancer: a descriptive correlational study. Support Care Cancer. (2021) 29(7):3697–705. 10.1007/s00520-020-05881-4 33190180

[24] Petersen CL. Spiritual care: minimizing the vulnerability of parents whose children with cancer face the end of life. J Pediatr Oncol Nurs. (2020) 37(2):105–15. 10.1177/1043454219887509 31718403

[25] Bekui BAA Aziato L Ohene LA Richter MS. Psychological and spiritual wellbeing of family caregivers of children with cancer at a teaching hospital in Ghana. Int J Afr Nurs Sci. (2020) 13:100231. 10.1016/j.ijans.2020.100231

[26] Puello-Alcocer EC Herrera-Espitia KK German-Orozco SC. Feelings and changes in children and adolescents with cancer in Montería (2019). Rev Cienc Cuid. (2020) 17(2):33–43. 10.22463/17949831.1756

[27] Nafratilova M Allenidekania A Wanda D. Still hoping for a miracle: parents’ experiences in caring for their child with cancer under palliative care. Indian J Palliat Care. (2018) 24(2):127–30. 10.4103/IJPC.IJPC_195_17 29736112 PMC5915876

[28] Vindrola-Padros C Brage E. What is not, but might be: the disnarrated in parents’ stories of their child’s cancer treatment. Soc Sci Med. (2017) 193:16–22. 10.1016/j.socscimed.2017.09.048 28992537

[29] Sisk BA Kang TI Mack JW. Sources of parental hope in pediatric oncology. Pediatr Blood Cancer. (2018) 65(6):e26981. 10.1002/pbc.26981 29369485

[30] Conway MF Pantaleao A Popp JM. Parents’ experience of hope when their child has cancer: perceived meaning and the influence of health care professionals. J Pediatr Oncol Nurs. (2017) 34(6):427–34. 10.1177/1043454217713454 28669252

[31] Kamihara J Nyborn JA Olcese ME Nickerson T Mack JW. Parental hope for children with advanced cancer. Pediatrics. (2015) 135(5):868–74. 10.1542/peds.2014-2855 25847801

[32] Santos S Crespo C Canavarro MC Kazak AE. Family rituals and quality of life in children with cancer and their parents: the role of family cohesion and hope. J Pediatr Psychol. (2015) 40(7):664–71. 10.1093/jpepsy/jsv013 25775914 PMC4542831

[33] Barrera M Granek L Shaheed J Nicholas D Beaune L D’Agostino NM. The tenacity and tenuousness of hope: parental experiences of hope when their child has a poor cancer prognosis. Cancer Nurs. (2013) 36(5):408–16. 10.1097/NCC.0b013e318291ba7d 23640272

[34] Granek L Barrera M Shaheed J Nicholas D Beaune L D'Agostino N. Trajectory of parental hope when a child has difficult-to-treat cancer: a prospective qualitative study. Psychooncology. (2013) 22(11):2436–44. 10.1002/pon.3305 23737306

[35] Chinchilla-Salcedo TC. What hope means to parents of children with cancer? Aquichan. (2013) 13(2):216–25. 10.5294/aqui.2013.13.2.7

[36] Wu LM Chin CC Haase JE Chen CH. Coping experiences of adolescents with cancer: a qualitative study. J Adv Nurs. (2009) 65(11):2358–66. 10.1111/j.1365-2648.2009.05097.x 19737322

[37] De Graves S Aranda S. Living with hope and fear—the uncertainty of childhood cancer after relapse. Cancer Nurs. (2008) 31(4):292–301. 10.1097/01.NCC.0000305745.41582.73 18600116

[38] Mack JW Wolfe J Cook EF Grier HE Cleary PD Weeks JC. Hope and prognostic disclosure. J Clin Oncol. (2007) 25(35):5636–42. 10.1200/JCO.2007.12.6110 18065734

[39] Kylmä J Juvakka T. Hope in parents of adolescents with cancer—factors endangering and engendering parental hope. Eur J Oncol Nurs. (2007) 11(3):262–71. 10.1016/j.ejon.2006.06.007 16962373

[40] Magão MTG Leal IP. Hope in parents of children with cancer: a phenomenological interpretative analysis of the relationship with health care professionals. Psic Saúde Doenças. (2001) 2(1):3–22.

[41] Fearnow-Kenney M Kliewer W. Threat appraisal and adjustment among children with cancer. J Psychosoc Oncol. (2000) 18(3):1–17. 10.1300/J077v18n03_01

[42] Garófalo CDC Silveira AO Wernet M Charepe Z Bally JMG Spurr S. Connecting suffering and hope: the experience of children and adolescents with cancer during hematopoietic stem cell transplantation. Clin Child Psychol Psychiatry. (2026) 31(1):265–80. 10.1177/13591045251390669 41137511

[43] Henriques NL Silva JB Charepe ZB Braga PP Duarte ED. Factors that promote and threaten hope in caregivers of children with chronic conditions. Rev Lat Am Enfermagem. (2023) 31:e3897. 10.1590/1518-8345.6366.3897 37194892 PMC10202231

[44] Ferraz A Faria S Jerónimo M Pereira MG. Parental psychological adjustment in pediatric acute lymphoblastic leukemia: the mediating role of family functioning and resilience. Cancers. (2025) 17(3):338. 10.3390/cancers17030338 39941710 PMC11816336

[45] Kübler-Ross E. On Death and Dying. New York: Macmillan (1969).

[46] Nanos SM Malakian A Foran L Klekovkina E Fitzgibbon KP Yu A. Psychosocial interventions to relieve traumatic stress symptoms in family caregivers of children with cancer: a scoping review. Psychooncology. (2025) 34(7):e70230. 10.1002/pon.70230 40671177 PMC12267684

[47] Marques-Mangolin EA Leite ACAB Alvarenga WA Neris RR Nunes MDR Nascimento LC. Exploring hope-centred care in paediatric chronic illness: a qualitative study of nurses’ perspectives. Collegian. (2026) 33:144–53. 10.1016/j.colegn.2026.02.002

[48] Ahmadi M Beiranvand S Haghighi S Akbari S. Predictive role of perceived social support and psychological distress on caregiver burden in parents of children with cancer. Semin Oncol Nurs. (2025) 41(3):151851. 10.1016/j.soncn.2025.151851 40187913

