# Supplementary material for: The hope of children and families in the context of cancer: a scoping review
Source: Front Pediatr. 2026 Oct 1;14:1958432. doi: 10.3389/fped.2026.1958432 (PMC13669171; doi:10.3389/fped.2026.1958432)
Supplement: Supplementary file 1 [file Table1.docx]

Supplementary Table 1: Complete search strategies for each database.

| Database | Number of articles | Search terms |
| --- | --- | --- |
| SCOPUS | 2191 | TITLE-ABS-KEY ((child OR children) AND (parents OR parenthood OR mothers OR fathers OR family OR families OR relatives OR siblings OR brother OR sister) AND (cancer OR neoplasms) AND (hope OR "coping strategies" OR "life experience" OR resiliency OR belief OR hopefulness OR "coping skills" OR "life change events" OR "psychological resilience")) |
| LILACS | 163 | ((child OR children) ) AND ((parents OR parenthood OR mothers OR fathers OR family OR families OR relatives OR siblings OR brother OR sister)) AND ((cancer OR neoplasms)) AND ((hope OR (coping strategies) OR (life experience) OR resiliency OR belief OR hopefulness OR (coping skills) OR (life change events) OR (resilience, psychological))) AND db:("LILACS") AND instance:"lilacsplus" |
| SCIELO | 224 | (ab:((child OR children))) AND (ab:((parents OR parenthood OR mothers OR fathers OR family OR families OR relatives OR siblings OR Brother OR sister))) AND (ab:((cancer OR neoplasms))) AND (ab:((hope OR (coping strategies) OR (life experience) OR resiliency OR belief OR Hopefulness OR (coping skills) OR (life change events) OR (Resilience, Psychological)))) |
| CINAHL | 761 | AB ((child OR children) AND (parents OR parenthood OR mothers OR fathers OR family OR families OR relatives OR siblings OR brother OR sister) AND (cancer OR neoplasms) AND (hope OR "coping strategies" OR "life experience" OR resiliency OR belief OR hopefulness OR "coping skills" OR "life change events" OR "psychological resilience")) |
| APA PsycINFO | 400 | (Abstract: child OR Abstract: children) AND (Abstract: parents OR Abstract: parenthood OR Abstract: mothers OR Abstract: fathers OR Abstract: family OR Abstract: families OR Abstract: relatives OR Abstract: siblings OR Abstract: Brother OR Abstract: sister) AND (Abstract: cancer OR Abstract: neoplasms) AND (Abstract: hope OR (Abstract: coping strategies) OR (Abstract: life experience) OR Abstract: resiliency OR Abstract: belief OR Abstract: Hopefulness OR (Abstract: coping skills) OR (Abstract: life change events) OR (Abstract: Resilience, Psychological)) |
| PUBMED | 641 | ("child"[Title/Abstract] OR "children"[Title/Abstract]) AND ("parents"[Title/Abstract] OR "parenthood"[Title/Abstract] OR "mothers"[Title/Abstract] OR "fathers"[Title/Abstract] OR "family"[Title/Abstract] OR "families"[Title/Abstract] OR "relatives"[Title/Abstract] OR "siblings"[Title/Abstract] OR "Brother"[Title/Abstract] OR "sister"[Title/Abstract]) AND ("cancer"[Title/Abstract] OR "neoplasms"[Title/Abstract]) AND ("hope"[Title/Abstract] OR "coping strategies"[Title/Abstract] OR "life experience"[Title/Abstract] OR "resiliency"[Title/Abstract] OR "belief"[Title/Abstract] OR "Hopefulness"[Title/Abstract] OR "coping skills"[Title/Abstract] OR "life change events"[Title/Abstract] OR "resilience psychological"[Title/Abstract]) |
